# Supplementary material for: Systems Biology Approach for Personalized Hemostasis Correction
Source: J Pers Med. 2022 Nov 15;12(11):1903. doi: 10.3390/jpm12111903 (PMC9694039; doi:10.3390/jpm12111903)
Supplement: Supplementary file 1 [file jpm-12-01903-s001.zip › jpm-2005464-supplementary.pdf]

Supplement

Detailed mechanism-driven model of blood coagulation

Table S1. Initial values

Parameter	Value
TF0	10^{-10} [mol/m^2]
VII0	10^{-5} [mol/m^3] × Dilution
VIIa0	10^{-7}[mol/m^3] × Dilution
X0	1.7×10^{-4}[mol/m^3] × Dilution
Xa0	0[mol/m^3]
II0	1.4×10^{-3} [mol/m^3] × Dilution
IIa0	0[mol/m^3]
Fg0	7.6×10^{-3}[mol/m^3] × Dilution
Fn0	0[mol/m^3]
IXa0	0[mol/m^3]
IX0	9×10^{-5}[mol/m^3] × Dilution
VIIIa0	0[mol/m^3]
VIII0	7×10^{-7}[mol/m^3] × Dilution
Va0	0[mol/m^3]
V0	2×10^{-5}[mol/m^3] × Dilution
XaTFPI0	0[mol/m^3]
TFPI0	0.25×10^{-6}[mol/m^3] × Dilution
AT0	3.4×10^{-3}[mol/m^3] × Dilution
XIa0	0[mol/m^3]
XI0	3×10^{-5}[mol/m^3] × Dilution
HC0	0[mol/m^3]
C1I	1.7×10^{-3}[mol/m^3] × Dilution
a2M0	3×10^{-3}[mol/m^3]
a1AT	4×10^{-2}[mol/m^3] × Dilution
a2AP	1.1×10^{-3}[mol/m^3] × Dilution
PCI	8.8×10^{-5}[mol/m^3] × Dilution
S0	0.4[mol/m^3]
TFden	0.8×10^{-10}[mol/m^2]
Dilution	0.67 – 1
PS0	1.31×10^{-4}[mol/m^3] × Dilution
PLi0	TF0 × PLi0_Coef
PLi0_Coef	5000
PLv0	0.2[mol/m^3] × Dilution
PLe0	0 - 4×10^{-3}[mol/m^3]

Table S2. Constants of reactions on the TF surface

Constant	Value
ka_VIIa_TF	2.6×10^{3} [m^3/(s×mol)]
ka_VII_TF	8.6×10^{2} [m^3/(s×mol)]
kd_VIIaTF	2.3×10^{-4} [1/s]

Constant	Value
kd_VIITF	2.3×10^{-4} [1/s]
kcat_VIITF_IIa	6.1×10^{-2} [1/s]
Ka_X_VIIaTF	10^{3} [m^3/(s×mol)]
Kd_XVIIaTF	0.5 [1/s]
kAct_VII_VIIaTF	434 [m^3/(s×mol)]
kAct_VIITF_Xa	935 [m^3/(s×mol)]
kAct_VIITF_VIIaTF	7.5×10^{6} [m^2/(s×mol)]
kAct_XVIIaTF	12 [1/s]
Ka_IX_VIIaTF	3×10^{4} [m^3/(s×mol)]
Kd_IXVIIaTF	0.45 [1/s]
Kact_IXVIIaTF	8.6 [1/s]
kAct_VIIa_VIITF	7.2 [m^3/(s×mol)]

Table S3. Reactions on the TF surface

Reaction	Expression	Unit
sVII_flux	− ka_VII_TF×VII×TF + kd_VIITF×VIITF − kAct_VII_VIIaTF×VII×VIIaTF	mol/(m^2×s)
sVIIa_flux	− ka_VIIa_TF×VIIa×TF + kd_VIIaTF×VIIaTF + kAct_VII_VIIaTF×VII×VIIaTF	mol/(m^2×s)
sVIIaTF_flux	ka_VIIa_TF×VIIa×TF − kd_VIIaTF×VIIaTF + kAct_VIITF_Xa×VIITF×Xa − ka_VIIaTF_XaTFPI×VIIaTF×XaTFPI + kd_VIIaTFXaTFPI×XaTFPIVIIaTF − Ka_X_VIIaTF×X×VIIaTF + Kd_XVIIaTF×XVIIaTF − Ka_IX_VIIaTF×IX×VIIaTF + Kd_IXVIIaTF×IXVIIaTF − Ki_VIIaTF_AT×VIIaTF×AT − Ki_VIIaTF_HCAT×VIIaTF×ATHC + kAct_VIIa_VIITF×VIIa×VIITF + kAct_XVIIaTF×XVIIaTF + Kact_IXVIIaTF×IXVIIaTF + kAct_VIITF_VIIaTF×VIIaTF×VIITF	mol/(m^2×s)
sVIITF_flux	ka_VII_TF×VII×TF − kd_VIITF×VIITF − kAct_VIITF_Xa×VIITF×Xa − kAct_VIITF_VIIaTF×VIIaTF×VIITF − kAct_VIIa_VIITF×VIIa×VIITF	mol/(m^2×s)
sX_flux	−Ka_X_VIIaTF×X×VIIaTF + Kd_XVIIaTF×XVIIaTF	mol/(m^2×s)
sXa_flux	kAct_XVIIaTF×XVIIaTF	mol/(m^2×s)
sTF_flux	−ka_VIIa_TF×VIIa×TF + kd_VIIaTF×VIIaTF −	mol/(m^2×s)

Reaction	Expression	Unit
	ka_VII_TF×VII×TF + kd_VIITF×VIITF	
sXVIIaTF_flux	Ka_X_VIIaTF×X×VIIaTF − Kd_XVIIaTF×XVIIaTF − kAct_XVIIaTF×XVIIaTF	mol/(m^2×s)
sXaTFPI_flux	−ka_VIIaTF_XaTFPI×XaTFPI×VIIaTF + kd_VIIaTFXaTFPI×XaTFPIVIIaTF	mol/(m^2×s)
sXaTFPIVIIaTF_flux	ka_VIIaTF_XaTFPI×XaTFPI×VIIaTF − kd_VIIaTFXaTFPI×XaTFPIVIIaTF	mol/(m^2×s)
sIXVIIaTF_flux	Ka_IX_VIIaTF×IX×VIIaTF − Kd_IXVIIaTF×IXVIIaTF − Kact_IXVIIaTF×IXVIIaTF	mol/(m^2×s)
sIX_flux	−Ka_IX_VIIaTF×IX×VIIaTF + Kd_IXVIIaTF×IXVIIaTF	mol/(m^2×s)
sIXa_flux	Kact_IXVIIaTF×IXVIIaTF	mol/(m^2×s)
sPL_flux	ni×(−ka_VII_TF×VII×TF + kd_VIITF×VIITF − ka_VIIa_TF×VIIa×TF + kd_VIIaTF×VIIaTF − Ka_X_VIIaTF×X×VIIaTF + Kd_XVIIaTF×XVIIaTF + kAct_XVIIaTF×XVIIaTF − ka_VIIaTF_XaTFPI×XaTFPI×VIIaTF + kd_VIIaTFXaTFPI×XaTFPIVIIaTF − Ka_IX_VIIaTF×IX×VIIaTF + Kd_IXVIIaTF×IXVIIaTF + Kact_IXVIIaTF×IXVIIaTF)	mol/(m^2×s)

Table S4. Inhibition constants

Constant	Value
ka_VIIaTF_XaTFPI	3.8×10^5 [m^3/(s×mol)]
ka_Xa_TFPI	9×10^4 [m^3/(s×mol)]
kd_XaTFPI	3.6×10^{-3} [1/s]
ka_IXa_ATIII	0.22 [m^3/(s×mol)]
ka_Xa_ATIII	2.5 [m^3/(s×mol)]
ka_Xa_a2M	1.4 [m^3/(s×mol)]
ka_Xa_a1AT	0.227 [m^3/(s×mol)]
ka_Xa_PCI	20 [m^3/(s×mol)]
ka_XaVaB_ATIII	0.14 [m^3/(s×mol)]
ka_IIa_ATIII	5.6 [m^3/(s×mol)]
ka_IIa_a2M	0.488 [m^3/(s×mol)]
ka_IIa_a1AT	0.1085 [m^3/(s×mol)]
ka_IIa_PCI	6.17 [m^3/(s×mol)]
ka_IIa_HCII	1.05 [m^3/(s×mol)]
ka_XIa_ATIII	8×10^{-3} [m^3/(s×mol)]
ka_XIa_a2AP	0.011 [m^3/(s×mol)]

Constant	Value
ka_XIa_a1AT	2.5×10^{-3} [m^3/(s×mol)]
ka_XIa_PCI	0.0225 [m^3/(s×mol)]
ka_XIa_C1I	6×10^{-3} [m^3/(s×mol)]
kd_VIIaTFXaTFPI	1×10^{-4} [1/s]
h_VIIIa	5.83×10^{-3} [1/s]
Ka_at3i	10 [m^3/(s×mol)]
Ki_VIIa_HCAT	0.17 [m^3/(s×mol)]
Ki_VIIaTF_AT	0.45 [m^3/(s×mol)]
Ki_VIIaTF_HCAT	5.6 [m^3/(s×mol)]
Kd_HCATweak	1.3 [1/s]
Ka_HC_ATweak	30 [m^3/(s×mol)]
Kcat_HCAT	440 [1/s]
Kcat_HCATweak	1.3 [1/s]
h_IXaVIIIa	1.7×10^{-4} [1/s]
ka_XaVaBi_ATIII	0.14 [m^2/(s×mol)]

Table S5. Constants of volume reactions

Constant	Value
Ka_Fg_IIa	4×10^{4} [m^3/(s×mol)]
Ka_Fn_IIa	2×10^{4} [m^3/(s×mol)]
Ka_S_IIa	15 [m^3/(s×mol)]
Ka_S_IIaA2M	6.1 [m^3/(s×mol)]
Ka_Xa_PS	5.5×10^{2} [m^3/(s×mol)]
kAct_VII_Xa	1.3×10^{4} [m^3/(s×mol)]
Kcat_FgIIa	84 [1/s]
Kcat_S_IIa	1.83 [1/s]
Kcat_S_IIaA2M	0.89 [1/s]
Kcat_XI_IIa	5×10^{-6} [1/s]
Kd_FgIIa	200 [1/s]
Kd_FnIIa	200 [1/s]
Kd_IIaA2MS	1 [1/s]
Kd_IIaS	2 [1/s]
Kd_XaPS	0.1 [1/s]
Keff_II_Xa	220 [m^3/(s×mol)]
Keff_IX_XIa	1×10^{4} [m^3/(s×mol)]
Keff_VIII_IIa	5×10^{3} [m^3/(s×mol)]
Keff_VIII_Xa	1.1×10^{3} [m^3/(s×mol)]
Keff_X_IXa	2.5 [m^3/(s×mol)]
Keff_XI_XIa	20 [m^3/(s×mol)]
KM_XI_IIa	5×10^{-5} [m^3/(s×mol)]

Table S6. Volume reactions

Reaction	Expression	Unit
a2M_flux	−(ka_IIa_a2M×a2M×IIa + ka_Xa_a2M×a2M×Xa)	mol/(m^3×s)

Reaction	Expression	Unit
AMC_flux	Kcat_S_IIa×IIaS + Kcat_S_IIaA2M×IIaa2MS	mol/(m^3×s)
AT_flux	−(ka_IXa_ATIII×IXa + ka_Xa_ATIII×Xa + ka_XaVaB_ATIII×XaVae+ka_XaVaB_ATIII×XaVav + ka_IIa_ATIII×IIa + ka_XIa_ATIII×XIa)×AT − AT×Ka_at3i×(IIaFg + IIaFn) − Ka_HC_ATweak×HC×AT + Kd_HCATweak×ATHCweak	mol/(m^3×s)
Fg_flux	−Ka_Fg_IIa×IIa×Fg + Kd_FgIIa×IIaFg + Ka_at3i×AT×IIaFg	mol/(m^3×s)
IIaFg_flux	Ka_Fg_IIa×IIa×Fg − Kd_FgIIa×IIaFg − Kcat_FgIIa×IIaFg − Ka_at3i×AT×IIaFg	mol/(m^3×s)
Fn_flux	−Ka_Fn_IIa×IIa×Fn + Kd_FnIIa×IIaFn + Ka_at3i×AT×IIaFn	mol/(m^3×s)
IIaFn_flux	Ka_Fn_IIa×IIa×Fn − Kd_FnIIa×IIaFn − Ka_at3i×AT×IIaFn + Kcat_FgIIa×IIaFg	mol/(m^3×s)
HC_flux	−Ka_HC_ATweak×HC×AT + Kd_HCATweak×ATHCweak	mol/(m^3×s)
ATHC_flux	Kcat_HCAT×ATHCweak − Kcat_HCATweak×ATHC	mol/(m^3×s)
ATHCweak_flux	Ka_HC_ATweak×HC×AT − Kd_HCATweak×ATHCweak − Kcat_HCAT×ATHCweak + Kcat_HCATweak×ATHC	mol/(m^3×s)
II_flux	−Keff_II_Xa×II×Xa	mol/(m^3×s)
IIa_flux	Keff_II_Xa×II×Xa − (ka_IIa_ATIII×AT + ka_IIa_a2M×a2M + ka_IIa_a1AT×a1AT + ka_IIa_PCI×PCI)×IIa − Ka_Fg_IIa×IIa×Fg + Kd_FgIIa×IIaFg − Ka_Fn_IIa×IIa×Fn + Kd_FnIIa×IIaFn − (Ka_S_IIa×S×IIa − Kd_IIaS×IIaS − Kcat_S_IIa×IIaS)	mol/(m^3×s)

Reaction	Expression	Unit
IIaa2M_flux	ka_IIa_a2M×a2M×IIa − Ka_S_IIaA2M×S×IIaa2M + Kd_IIaA2MS×IIaa2MS + Kcat_S_IIaA2M×IIaa2MS	mol/(m^3×s)
IIaa2MS_flux	Ka_S_IIaA2M×S×IIaa2M − Kd_IIaA2MS×IIaa2MS − Kcat_S_IIaA2M×IIaa2MS	mol/(m^3×s)
IIaS_flux	Ka_S_IIa×S×IIa − Kd_IIaS×IIaS − Kcat_S_IIa×IIaS	mol/(m^3×s)
IX_flux	−Keff_IX_XIa×IX×XIa	mol/(m^3×s)
IXa_flux	Keff_IX_XIa×IX×XIa − ka_IXa_ATIII×AT×IXa	mol/(m^3×s)
PS_flux	−Ka_Xa_PS×Xa×PS + Kd_XaPS×XaPS − Ka_Xa_PS×X×PS + Kd_XaPS×XPS	mol/(m^3×s)
S_flux	−Ka_S_IIa×S×IIa + Kd_IIaS×IIaS − Ka_S_IIaA2M×S×IIaa2M + Kd_IIaA2MS×IIaa2MS	mol/(m^3×s)
TFPI_flux	−ka_Xa_TFPI×Xa×TFPI + kd_XaTFPI×XaTFPI	mol/(m^3×s)
V_flux	0	
Va_flux	0	
VII_flux	−kAct_VII_Xa×VII×Xa	mol/(m^3×s)
VIIa_flux	kAct_VII_Xa×VII×Xa	mol/(m^3×s)
VIII_flux	0	
VIIIa_flux	−h_VIIIa×VIIIa	mol/(m^3×s)
X_flux	−Keff_X_IXa×X×IXa − Ka_Xa_PS×X×PS + Kd_XaPS×XPS	mol/(m^3×s)
Xa_flux	Keff_X_IXa×X×IXa − Ka_Xa_PS×Xa×PS + Kd_XaPS×XaPS − (ka_Xa_ATIII×AT + ka_Xa_a2M×a2M + ka_Xa_a1AT×a1AT + ka_Xa_PCI×PCI)×Xa − ka_Xa_TFPI×Xa×TFPI + kd_XaTFPI×XaTFPI	mol/(m^3×s)
XaPS_flux	Ka_Xa_PS×Xa×PS − Kd_XaPS×XaPS	mol/(m^3×s)
XPS_flux	Ka_Xa_PS×X×PS − Kd_XaPS×XPS	mol/(m^3×s)
XaTFPI_flux	ka_Xa_TFPI×Xa×TFPI − kd_XaTFPI×XaTFPI	mol/(m^3×s)

Reaction	Expression	Unit
XI_flux	−Kcat_XI_IIa×XI×(IIa)/(KM_XI_IIa + XI) − Keff_XI_XIa×XI×XIa	mol/(m^3×s)
XIa_flux	Kcat_XI_IIa×XI×(IIa)/(KM_XI_IIa + XI) − (ka_XIa_ATIII×AT + ka_XIa_a2AP×a2AP + ka_XIa_a1AT×a1AT + ka_XIa_PCI×PCI + ka_XIa_C1I×C1I)×XIa + Keff_XI_XIa×XI×XIa	mol/(m^3×s)

Table S7. Constants of reactions on the TF-containing lipids

Constant	Value
Ka_II_PLi	15 [m^3/(s×mol)]
Ka_II_XaVai	4.1×10^4 [m^3/(s×mol)]
Ka_IXa_VIIIaBi	(1 + X0/1×10^{-5}[mol/m^3]) ×2.5×10^4 [m^3/(s×mol)]
Ka_PS_VaBi	1.3×10^4 [m^3/(s×mol)]
Ka_V_PLi	740 [m^3/(s×mol)]
Ka_Va_PLi	740 [m^3/(s×mol)]
Ka_VIII_PLi	630 [m^3/(s×mol)]
Ka_VIIIa_PLi	780 [m^3/(s×mol)]
Ka_X_IXaVIIIai	1.8×10^5 [m^3/(s×mol)]
Ka_Xa_VaBi	4440 [m^3/(s×mol)]
Ka_XI_VaBi	35 [m^3/(s×mol)]
Ka_XI_VBi	37.5 [m^3/(s×mol)]
Kcat_IIXaVai	108 [1/s]
Kcat_VBi_IIa	2.3 [1/s]
Kcat_VBi_Xa	4.3 [1/s]
Kcat_X_IXaVIIIai	5 [1/s]
Kd_IIBi	2×10^{-3} [1/s]
Kd_IIXaVai	10 [1/s]
Kd_IXaVIIIai	2.2×10^{-3} [1/s]
Kd_PSVaBi	0.1 [1/s]
Kd_VaBi	2×10^{-3} [1/s]
Kd_VBi	5×10^{-3} [1/s]
Kd_VIIIaBi	2.8×10^{-4} [1/s]
Kd_VIIIBi	1.3×10^{-3} [1/s]
Kd_XaVai	2×10^{-3} [1/s]
Kd_XIVaBi	1.5×10^{-4} [1/s]
Kd_XIVBi	1.8×10^{-4} [1/s]
Kd_XIXaVIIIai	10 [1/s]
Keff_XIVBi_IIa	8 [m^3/(s×mol)]
KM_VBi_IIa	7.17×10^{-5} [mol/m^3]
KM_VBi_Xa	1.04×10^{-5} [mol/m^3]

Constant	Value
ni	100
Ka_IIBi_XaVai	4.1×10^4 [m^2/(s×mol)]

Table S8. Reactions on the TF-containing lipids

Reaction	Expression	Unit
II_i_flux	−Ka_II_PLi×II×PLi + Kd_IIBi×IIBi − Ka_II_XaVai×II×XaVai + Kd_IIXaVai×IIXaVai	mol/(m^2×s)
IIa_i_flux	Kcat_IIXaVai×IIXaVai	mol/(m^2×s)
IIBi_flux	Ka_II_PLi×II×PLi − Kd_IIBi×IIBi − Ka_IIBi_XaVai×IIBi×XaVai	mol/(m^2×s)
IXa_i_flux	−Ka_IXa_VIIIaBi×IXa×VIIIaBi + Kd_IXaVIIIai×IXaVIIIai + h_IXaVIIIa×IXaVIIIai	mol/(m^2×s)
IXaVIIIai_flux	Ka_IXa_VIIIaBi×IXa×VIIIaBi − Kd_IXaVIIIai×IXaVIIIai − h_IXaVIIIa×IXaVIIIai − Ka_X_IXaVIIIai×X×IXaVIIIai + Kd_XIXaVIIIai×XIXaVIIIai	mol/(m^2×s)
PS_i_flux	−Ka_PS_VaBi×PS×VaBi + Kd_PSVaBi×PSVai	mol/(m^2×s)
PSVai_flux	Ka_PS_VaBi×PS×VaBi − Kd_PSVaBi×PSVai	mol/(m^2×s)
V_i_flux	−Ka_V_PLi×V×PLi + Kd_VBi×VBi	mol/(m^2×s)
Va_i_flux	−Ka_Va_PLi×Va×PLi + Kd_VaBi×VaBi	mol/(m^2×s)
VaBi_flux	−Ka_PS_VaBi×PS×VaBi + Kd_PSVaBi×PSVai + Ka_Va_PLi×Va×PLi − Kd_VaBi×VaBi − Ka_Xa_VaBi×Xa×VaBi + Kd_XaVai×XaVai + Kcat_VBi_Xa×VBi×Xa/(KM_VBi_Xa + VBi×1[1/m]) + Kcat_VBi_IIa×VBi×(IIa)/(KM_VBi_IIa + VBi×1[1/m]) − Ka_XI_VaBi×XI×VaBi + Kd_XIVaBi×XIVaBi + Keff_XIVBi_IIa×XIVaBi×IIa	mol/(m^2×s)
VBi_flux	Ka_V_PLi×V×PLi − Kd_VBi×VBi − Kcat_VBi_Xa×VBi×Xa/(KM_VBi_Xa + VBi×1[1/m]) − Kcat_VBi_IIa×VBi×(IIa)/(KM_VBi_IIa + VBi×1[1/m]) − Ka_XI_VBi×XI×VBi + Kd_XIVBi×XIVBi + Keff_XIVBi_IIa×XIVBi×IIa	mol/(m^2×s)
VIII_i_flux	−Ka_VIII_PLi×VIII×PLi + Kd_VIIIBi×VIIIBi	mol/(m^2×s)
VIIIa_i_flux	−Ka_VIIIa_PLi×VIIIa×PLi + Kd_VIIIaBi×VIIIaBi	mol/(m^2×s)

Reaction	Expression	Unit
VIIIaBi_flux	Ka_VIIIa_PLi×VIIIa×PLi − Kd_VIIIaBi×VIIIaBi − h_VIIIa×VIIIaBi + Keff_VIII_IIa×VIIIBi×(IIa) + Keff_VIII_Xa×VIIIBi×(Xa) − Ka_IXa_VIIIaBi×(IXa)×VIIIaBi + Kd_IXaVIIIai×IXaVIIIai	$mol/(m^2×s)$
VIIIBi_flux	Ka_VIII_PLi×VIII×PLi − Kd_VIIIBi×VIIIBi − Keff_VIII_IIa×VIIIBi×(IIa) − Keff_VIII_Xa×VIIIBi×(Xa)	$mol/(m^2×s)$
X_i_flux	−Ka_X_IXaVIIIai×X×IXaVIIIai + Kd_XIXaVIIIai×XIXaVIIIai	$mol/(m^2×s)$
Xa_i_flux	−Ka_Xa_VaBi×Xa×VaBi + Kd_XaVai×XaVai + Kcat_X_IXaVIIIai×XIXaVIIIai	$mol/(m^2×s)$
XaVai_flux	Ka_Xa_VaBi×(Xa)×VaBi − Kd_XaVai×XaVai − ka_XaVaB_ATIII×XaVai×AT − Ka_II_XaVai×II×XaVai + Kd_IIXaVai×IIXaVai + Kcat_IIXaVai×IIXaVai	$mol/(m^2×s)$
XIVaBi_flux	Ka_XI_VaBi×XI×VaBi − Kd_XIVaBi×XIVaBi − Keff_XIVBi_IIa×XIVaBi×(IIa)	$mol/(m^2×s)$
XIVBi_flux	Ka_XI_VBi×XI×VBi − Kd_XIVBi×XIVBi − Keff_XIVBi_IIa×XIVBi×(IIa)	$mol/(m^2×s)$
XIXaVIIIai_flux	Ka_X_IXaVIIIai×(X)×IXaVIIIai − Kd_XIXaVIIIai×XIXaVIIIai − Kcat_X_IXaVIIIai×XIXaVIIIai	$mol/(m^2×s)$
IIXaVai_flux	Ka_II_XaVai×II×XaVai − Kd_IIXaVai×IIXaVai − Kcat_IIXaVai×IIXaVai	$mol/(m^2×s)$
PLi_flux	ni×(−Ka_II_PLi×II×PLi + Kd_IIBi×IIBi − Ka_II_XaVai×II×XaVai + Kd_IIXaVai×IIXaVai + Kcat_IIXaVai×IIXaVai + h_IXaVIIIa×IXaVIIIai − Ka_PS_VaBi×PS×VaBi + Kd_PSVaBi×PSVai − Ka_V_PLi×V×PLi + Kd_VBi×VBi − Ka_Va_PLi×Va×PLi + Kd_VaBi×VaBi − Ka_Xa_VaBi×Xa×VaBi + Kd_XaVai×XaVai + ka_XaVaB_ATIII×XaVai×AT − Ka_XI_VaBi×XI×VaBi + Kd_XIVaBi×XIVaBi + Keff_XIVBi_IIa×XIVaBi×IIa −	$mol/(m^2×s)$

Reaction	Expression	Unit
	Ka_XI_VBi×XI×VBi + Kd_XIVBi×XIVBi + Keff_XIVBi_IIa×XIVBi×IIa – Ka_VIII_PLi×VIII×PLi + Kd_VIIIBi×VIIIBi – Ka_VIIIa_PLi×VIIIa×PLi + Kd_VIIIaBi×VIIIaBi + h_VIIIa×VIIIaBi – Ka_IXa_VIIIaBi×(IXa)×VIIIaBi + Kd_IXaVIIIai×IXaVIIIai – Ka_X_IXaVIIIai×X×IXaVIIIai + Kd_XIXaVIIIai×XIXaVIIIai + Kcat_X_IXaVIIIai×XIXaVIIIai)	
XI_i_flux	−Ka_XI_VaBi×XI×VaBi + Kd_XIVaBi×XIVaBi – Ka_XI_VaBi×XI×VaBi + Kd_XIVaBi×XIVaBi	mol/(m^2×s)
XIa_i_flux	Keff_XIVBi_IIa×XIVaBi×(IIa) + Keff_XIVBi_IIa×XIVBi×(IIa)	mol/(m^2×s)

Table S9. Constants of reactions on the exogenous lipids

Constant	Value
Ka_II_PLe	15 [m^3/(s×mol)]
Ka_II_XaVae	4.1×10^4 [m^3/(s×mol)]
Ka_IXa_VIIIaBe	1*(1 + X0/1×10^{-5}[mol/m^3]) ×2.5×10^4 [m^3/(s×mol)]
Ka_PS_VaBe	1.3×10^4 [m^3/(s×mol)]
Ka_V_PLe	740 [m^3/(s×mol)]
Ka_Va_PLe	740 [m^3/(s×mol)]
Ka_VIII_PLe	630 [m^3/(s×mol)]
Ka_VIIIa_PLe	780 [m^3/(s×mol)]
Ka_X_IXaVIIIae	1.8×10^5 [m^3/(s×mol)]
Ka_Xa_VaBe	4440 [m^3/(s×mol)]
Ka_XI_VaBe	35 [m^3/(s×mol)]
Ka_XI_VBe	37.5 [m^3/(s×mol)]
Kcat_IIXaVae	54 [1/s]
Kcat_VBe_IIa	2.3 [1/s]
Kcat_VBe_Xa	4.3 [1/s]
Kcat_X_IXaVIIIae	5 [1/s]
Kd_IIBe	2×10^{-3} [1/s]
Kd_IIXaVae	10 [1/s]
Kd_IXaVIIIae	2.2×10^{-3} [1/s]
Kd_PSVaBe	0.1 [1/s]
Kd_VaBe	2×10^{-3} [1/s]
Kd_VBe	5×10^{-3} [1/s]
Kd_VIIIaBe	2.8×10^{-4} [1/s]
Kd_VIIIBe	1.3×10^{-3} [1/s]
Kd_XaVae	2×10^{-3} [1/s]

Constant	Value
Kd_XIVaBe	1.5×10^{-4} [1/s]
Kd_XIVBe	1.8×10^{-4} [1/s]
Kd_XIXaVIIIae	1 [1/s]
Keff_XIVBe_IIa	8 [m^3/(s×mol)]
KM_VBe_IIa	7.17×10^{-5} [mol/m^3]
KM_VBe_Xa	1.04×10^{-5}[mol/m^3]
ne	100

Table S10. Reactions on the exogenous lipids

Reaction	Expression	Unit
II_e_flux	−Ka_II_PLe×II×PLe+Kd_IIBe×IIBe−Ka_II_XaVae×II×XaVae+Kd_IIXaVae×IIXaVae	mol/(m^3×s)
IIa_e_flux	Kcat_IIXaVae×IIXaVae	mol/(m^3×s)
IIBe_flux	Ka_II_PLe×II×PLe−Kd_IIBe×IIBe−Ka_II_XaVae×IIBe×XaVae	mol/(m^3×s)
IXa_e_flux	−Ka_IXa_VIIIaBe×IXa×VIIIaBe+Kd_IXaVIIIae×IXaVIIIae+h_IXaVIIIa×IXaVIIIae	mol/(m^3×s)
IXaVIIIae_flux	Ka_IXa_VIIIaBe×IXa×VIIIaBe−Kd_IXaVIIIae×IXaVIIIae−h_IXaVIIIa×IXaVIIIae−Ka_X_IXaVIIIae×X×IXaVIIIae+Kd_XIXaVIIIae×XIXaVIIIae	mol/(m^3×s)
PS_e_flux	−Ka_PS_VaBe×PS×VaBe+Kd_PSVaBe×PSVae	mol/(m^3×s)
PSVae_flux	Ka_PS_VaBe×PS×VaBe−Kd_PSVaBe×PSVae	mol/(m^3×s)
V_e_flux	−Ka_V_PLe×V×PLe+Kd_VBe×VBe	mol/(m^3×s)
Va_e_flux	−Ka_Va_PLe×Va×PLe+Kd_VaBe×VaBe	mol/(m^3×s)
VaBe_flux	−Ka_PS_VaBe×PS×VaBe+Kd_PSVaBe×PSVae+Ka_Va_PLe×Va×PLe−Kd_VaBe×VaBe−Ka_Xa_VaBe×Xa×VaBe+Kd_XaVae×XaVae+Kcat_VBe_Xa×VBe×(Xa)/(KM_VBe_Xa + VBe) + Kcat_VBe_IIa×VBe×(IIa)/(KM_VBe_IIa + VBe) − Ka_XI_VaBe×XI×VaBe+Kd_XIVaBe×XIVaBe+Keff_XIVBe_IIa×XIVaBe×IIa	mol/(m^3×s)
VBe_flux	Ka_V_PLe×V×PLe−Kd_VBe×VBe−Kcat_VBe_Xa×VBe×Xa/(KM_VBe_Xa + VBe) − Kcat_VBe_IIa×VBe×(IIa)/(KM_VBe_IIa + VBe) − Ka_XI_VBe×XI×VBe+Kd_XIVBe×XIVBe+Keff_XIVBe_IIa×XIVBe×IIa	mol/(m^3×s)
VIII_e_flux	−Ka_VIII_PLe×VIII×PLe+Kd_VIIIBe×VIIIBe	mol/(m^3×s)

Reaction	Expression	Unit
VIIIa_e_flux	−Ka_VIIIa_PLe×VIIIa×PLe+Kd_VIIIaBe ×VIIIaBe	mol/(m^3×s)
VIIIaBe_flux	Ka_VIIIa_PLe×VIIIa×PLe−Kd_VIIIaBe× VIIIaBe−h_VIIIa×VIIIaBe+Keff_VIII_IIa ×VIIIBe×(IIa) + Keff_VIII_Xa×VIIIBe×(Xa) − Ka_IXa_VIIIaBe×(IXa)×VIIIaBe+Kd_IXa VIIIae×IXaVIIIae	mol/(m^3×s)
VIIIBe_flux	Ka_VIII_PLe×VIII×PLe−Kd_VIIIBe×VIII Be−Keff_VIII_IIa×VIIIBe×(IIa) − Keff_VIII_Xa×VIIIBe×(Xa)	mol/(m^3×s)
X_e_flux	−Ka_X_IXaVIIIae×X×IXaVIIIae+Kd_XI XaVIIIae×XIXaVIIIae	mol/(m^3×s)
Xa_e_flux	−Ka_Xa_VaBe×Xa×VaBe+Kd_XaVae×X aVae+Kcat_X_IXaVIIIae×XIXaVIIIae	mol/(m^3×s)
XaVae_flux	Ka_Xa_VaBe×(Xa)×VaBe−Kd_XaVae×X aVae−ka_XaVaB_ATIII×XaVae×AT − Ka_II_XaVae×II×XaVae+Kd_IIXaVae×II XaVae+Kcat_IIXaVae×IIXaVae	mol/(m^3×s)
XIVaBe_flux	Ka_XI_VaBe×XI×VaBe−Kd_XIVaBe×XI VaBe−Keff_XIVBe_IIa×XIVaBe×(IIa)	mol/(m^3×s)
XIVBe_flux	Ka_XI_VBe×XI×VBe−Kd_XIVBe×XIVB e−Keff_XIVBe_IIa×XIVBe×(IIa)	mol/(m^3×s)
XIXaVIIIae_flux	Ka_X_IXaVIIIae×(X)×IXaVIIIae−Kd_XI XaVIIIae×XIXaVIIIae−Kcat_X_IXaVIIIae ×XIXaVIIIae	mol/(m^3*s)
IIXaVae_flux	Ka_II_XaVae×II×XaVae−Kd_IIXaVae×II XaVae−Kcat_IIXaVae×IIXaVae	mol/(m^3×s)
PLe_flux	ne×(−Ka_II_PLe×II×PLe+Kd_IIBe×IIBe− Ka_II_XaVae×II×XaVae+Kd_IIXaVae×II XaVae+Kcat_IIXaVae×IIXaVae+h_IXaVI IIa×IXaVIIIae−Ka_PS_VaBe×PS×VaBe+ Kd_PSVaBe×PSVae−Ka_V_PLe×V×PLe+ Kd_VBe×VBe−Ka_Va_PLe×Va×PLe+Kd _VaBe×VaBe−Ka_Xa_VaBe×Xa×VaBe+ Kd_XaVae×XaVae+ka_XaVaB_ATIII×Xa Vae×AT − Ka_XI_VaBe×XI×VaBe+Kd_XIVaBe×XI VaBe+Keff_XIVBe_IIa×XIVaBe×IIa − Ka_XI_VBe×XI×VBe+Kd_XIVBe×XIVB e+Keff_XIVBe_IIa×XIVBe×IIa − Ka_VIII_PLe×VIII×PLe+Kd_VIIIBe×VIII Be−Ka_VIIIa_PLe×VIIIa×PLe+Kd_VIIIa Be×VIIIaBe+h_VIIIa×VIIIaBe−Ka_IXa_V IIIaBe×(IXa)×VIIIaBe+Kd_IXaVIIIae×IX aVIIIae−Ka_X_IXaVIIIae×X×IXaVIIIae+	mol/(m^3×s)

Reaction	Expression	Unit
	Kd_XIXaVIIIae×XIXaVIIIae+Kcat_X_IXaVIIIae×XIXaVIIIae)	
XI_e_flux	−Ka_XI_VaBe×XI×VaBe+Kd_XIVaBe×XIVaBe−Ka_XI_VaBe×XI×VaBe+Kd_XIVaBe×XIVaBe	mol/(m^3×s)
XIa_e_flux	Keff_XIVBe_IIa×XIVaBe×(IIa) + Keff_XIVBe_IIa×XIVBe×(IIa)	mol/(m^3×s)

Table S11. Constants of reactions on the endogenous lipids

Constant	Value
Ka_II_PLv	1.5×10^{-3} [m^3/(s×mol)]
Ka_II_XaVav	4.1×10^4 [m^3/(s×mol)]
Ka_IXa_VIIIaBv	(1 + X0/1×10^{-5}[mol/m^3]) ×2.5×10^3 [m^3/(s×mol)]
Ka_PS_VaBv	1.3×10^4 [m^3/(s×mol)]
Ka_V_PLv	7.4×10^{-2} [m^3/(s×mol)]
Ka_Va_PLv	7.4×10^{-2} [m^3/(s×mol)]
Ka_VIII_PLv	6.3×10^{-2} [m^3/(s×mol)]
Ka_VIIIa_PLv	7.8×10^{-2} [m^3/(s×mol)]
Ka_X_IXaVIIIav	1.8×10^5 [m^3/(s×mol)]
Ka_Xa_VaBv	444 [m^3/(s×mol)]
Ka_XI_VaBv	35 [m^3/(s×mol)]
Ka_XI_VBv	37.5 [m^3/(s×mol)]
Kcat_IIXaVav	10.8 [1/s]
Kcat_VBv_IIa	0.23 [1/s]
Kcat_VBv_Xa	0.043 [1/s]
Kcat_X_IXaVIIIav	5 [1/s]
Kd_IIBv	2×10^{-3} [1/s]
Kd_IIXaVav	10 [1/s]
Kd_IXaVIIIav	2.2×10^{-3} [1/s]
Kd_PSVaBv	0.1 [1/s]
Kd_VaBv	2×10^{-3} [1/s]
Kd_VBv	5×10^{-3} [1/s]
Kd_VIIIaBv	2.8×10^{-4} [1/s]
Kd_VIIIBv	1.3×10^{-3} [1/s]
Kd_XaVav	2×10^{-3} [1/s]
Kd_XIVaBv	1.5×10^{-4} [1/s]
Kd_XIVBv	1.8×10^{-4} [1/s]
Kd_XIXaVIIIav	10 [1/s]
Keff_XIVBv_IIa	0
KM_VBv_IIa	7.17×10^{-5} [mol/m^3]
KM_VBv_Xa	1.04×10^{-5} [mol/m^3]
nv	100

Table S12. Reactions on the endogenous lipids

**Reaction**	**Expression**	**Unit**
II_v_flux	−Ka_II_PLv×II×PLv + Kd_IIBv×IIBv − Ka_II_XaVav×II×XaVav + Kd_IIXaVav×IIXaVav	mol/(m^3×s)
IIa_v_flux	Kcat_IIXaVav×IIXaVav	mol/(m^3×s)
IIBv_flux	Ka_II_PLv×II×PLv − Kd_IIBv×IIBv − Ka_II_XaVav×IIBv×XaVav	mol/(m^3×s)
IXa_v_flux	−Ka_IXa_VIIIaBv×IXa×VIIIaBv + Kd_IXaVIIIav×IXaVIIIav + h_IXaVIIIa×IXaVIIIav	mol/(m^3×s)
IXaVIIIav_flux	Ka_IXa_VIIIaBv×IXa×VIIIaBv − Kd_IXaVIIIav×IXaVIIIav − h_IXaVIIIa×IXaVIIIav − Ka_X_IXaVIIIav×X×IXaVIIIav + Kd_XIXaVIIIav×XIXaVIIIav	mol/(m^3×s)
PS_v_flux	−Ka_PS_VaBv×PS×VaBv + Kd_PSVaBv×PSVav	mol/(m^3×s)
PSVav_flux	Ka_PS_VaBv×PS×VaBv − Kd_PSVaBv×PSVav	mol/(m^3×s)
V_v_flux	−Ka_V_PLv×V×PLv + Kd_VBv×VBv	mol/(m^3×s)
Va_v_flux	−Ka_Va_PLv×Va×PLv + Kd_VaBv×VaBv	mol/(m^3×s)
VaBv_flux	−Ka_PS_VaBv×PS×VaBv + Kd_PSVaBv×PSVav + Ka_Va_PLv×Va×PLv − Kd_VaBv×VaBv − Ka_Xa_VaBv×Xa×VaBv + Kd_XaVav×XaVav + Kcat_VBv_Xa×VBv×(Xa)/(KM_VBv_Xa + VBv) + Kcat_VBv_IIa×VBv×(IIa)/(KM_VBv_IIa + VBv) − Ka_XI_VaBv×XI×VaBv + Kd_XIVaBv×XIVaBv + Keff_XIVBv_IIa×XIVaBv×IIa	mol/(m^3×s)
VBv_flux	Ka_V_PLv×V×PLv − Kd_VBv×VBv − Kcat_VBv_Xa×VBv×Xa/(KM_VBv_Xa + VBv) − Kcat_VBv_IIa×VBv×(IIa)/(KM_VBv_IIa + VBv) − Ka_XI_VBv×XI×VBv + Kd_XIVBv×XIVBv + Keff_XIVBv_IIa×XIVBv×IIa	mol/(m^3×s)
VIII_v_flux	−Ka_VIII_PLv×VIII×PLv + Kd_VIIIBv×VIIIBv	mol/(m^3×s)
VIIIa_v_flux	−Ka_VIIIa_PLv×VIIIa×PLv + Kd_VIIIaBv×VIIIaBv	mol/(m^3×s)
VIIIaBv_flux	Ka_VIIIa_PLv×VIIIa×PLv − Kd_VIIIaBv×VIIIaBv − h_VIIIa×VIIIaBv + Keff_VIII_IIa×VIIIBv×(IIa) +	mol/(m^3×s)

Reaction	Expression	Unit
	Keff_VIII_Xa×VIIIBv×(Xa) − Ka_IXa_VIIIaBv×(IXa)×VIIIaBv + Kd_IXaVIIIav×IXaVIIIav	
VIIIBv_flux	Ka_VIII_PLv×VIII×PLv − Kd_VIIIBv×VIIIBv − Keff_VIII_IIa×VIIIBv×(IIa) − Keff_VIII_Xa×VIIIBv×(Xa)	mol/(m^3×s)
X_v_flux	−Ka_X_IXaVIIIav×X×IXaVIIIav + Kd_XIXaVIIIav×XIXaVIIIav	mol/(m^3×s)
Xa_v_flux	−Ka_Xa_VaBv×Xa×VaBv + Kd_XaVav×XaVav + Kcat_X_IXaVIIIav×XIXaVIIIav	mol/(m^3×s)
XaVav_flux	Ka_Xa_VaBv×(Xa)×VaBv − Kd_XaVav×XaVav − ka_XaVaB_ATIII×XaVav×AT − Ka_II_XaVav×II×XaVav + Kd_IIXaVav×IIXaVav + Kcat_IIXaVav×IIXaVav	mol/(m^3×s)
XIVaBv_flux	Ka_XI_VaBv×XI×VaBv − Kd_XIVaBv×XIVaBv − Keff_XIVBv_IIa×XIVaBv×(IIa)	mol/(m^3×s)
XIVBv_flux	Ka_XI_VBv×XI×VBv − Kd_XIVBv×XIVBv − Keff_XIVBv_IIa×XIVBv×(IIa)	mol/(m^3×s)
XIXaVIIIav_flux	Ka_X_IXaVIIIav×(X)×IXaVIIIav − Kd_XIXaVIIIav×XIXaVIIIav − Kcat_X_IXaVIIIav×XIXaVIIIav	mol/(m^3×s)
IIXaVav_flux	Ka_II_XaVav×II×XaVav − Kd_IIXaVav×IIXaVav − Kcat_IIXaVav×IIXaVav	mol/(m^3×s)
PLv_flux	nv×(−Ka_II_PLv×II×PLv + Kd_IIBv×IIBv − Ka_II_XaVav×II×XaVav + Kd_IIXaVav×IIXaVav + Kcat_IIXaVav×IIXaVav + h_IXaVIIIa×IXaVIIIav − Ka_PS_VaBv×PS×VaBv + Kd_PSVaBv×PSVav − Ka_V_PLv×V×PLv + Kd_VBv×VBv − Ka_Va_PLv×Va×PLv + Kd_VaBv×VaBv − Ka_Xa_VaBv×Xa×VaBv + Kd_XaVav×XaVav + ka_XaVaB_ATIII×XaVav×AT − Ka_XI_VaBv×XI×VaBv + Kd_XIVaBv×XIVaBv + Keff_XIVBv_IIa×XIVaBv×IIa − Ka_XI_VBv×XI×VBv + Kd_XIVBv×XIVBv +	mol/(m^3×s)

Reaction	Expression	Unit
	Keff_XIVBv_IIa×XIVBv×IIa − Ka_VIII_PLv×VIII×PLv + Kd_VIIIBv×VIIIBv − Ka_VIIIa_PLv×VIIIa×PLv + Kd_VIIIaBv×VIIIaBv + h_VIIIa×VIIIaBv − Ka_IXa_VIIIaBv×(IXa)×VIIIaBv + Kd_IXaVIIIav×IXaVIIIav − Ka_X_IXaVIIIav×X×IXaVIIIav + Kd_XIXaVIIIav×XIXaVIIIav + Kcat_X_IXaVIIIav×XIXaVIIIav)	
XI_v_flux	−Ka_XI_VaBv×XI×VaBv + Kd_XIVaBv×XIVaBv − Ka_XI_VaBv×XI×VaBv + Kd_XIVaBv×XIVaBv	mol/(m^3×s)
XIa_v_flux	Keff_XIVBv_IIa×XIVaBv×(IIa) + Keff_XIVBv_IIa×XIVBv×(IIa)	mol/(m^3×s)

Table S13. Diffusion constants

Constant	Value
D_AMC	3.5×10^{-10} [m^2/s]
D_AT	5.5×10^{-11} [m^2/s]
D_Fg	2×10^{-11} [m^2/s]
D_Fn	0 [m^2/s]
D_HC	8.8×10^{-11} [m^2/s]
D_ATHC	5×10^{-11} [m^2/s]
D_II	5×10^{-11} [m^2/s]
D_IIa	6.67×10^{-11} [m^2/s]
D_IX	5.5×10^{-11} [m^2/s]
D_IXa	6.17×10^{-11} [m^2/s]
D_PS	5×10^{-11} [m^2/s]
D_TFPI	6.5×10^{-11} [m^2/s]
D_V	2.67×10^{-11} [m^2/s]
D_Va	3.67×10^{-11} [m^2/s]
D_VII	5.83×10^{-11} [m^2/s]
D_VIIa	5.83×10^{-11} [m^2/s]
D_VIII	0 [m^2/s]
D_VIIIa	3.5×10^{-11} [m^2/s]
D_X	5.5×10^{-11} [m^2/s]
D_Xa	6.17×10^{-11} [m^2/s]
D_XaPS	4.2×10^{-11} [m^2/s]
D_XaTFPI	4.5×10^{-11} [m^2/s]
D_XI	3.5×10^{-11} [m^2/s]
D_XIa	3.5×10^{-11} [m^2/s]
D_a2M	1×10^{-11} [m^2/s]

Geometry of simulation area

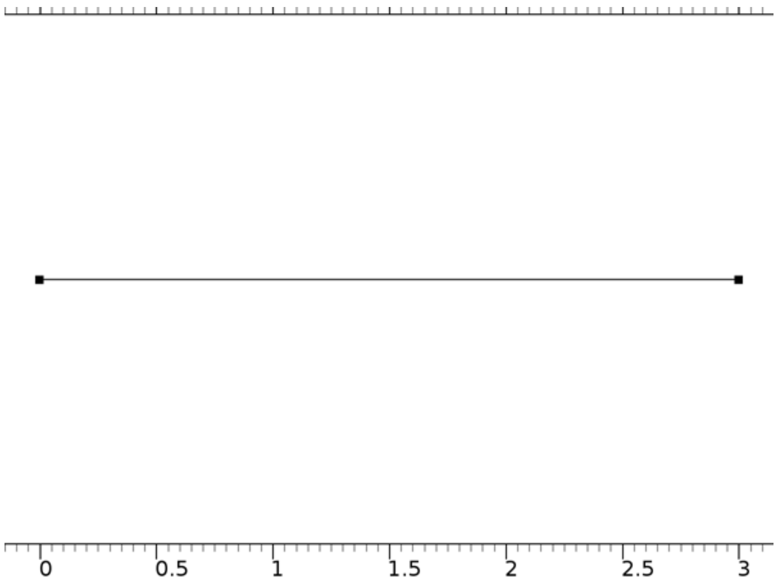

Units: mm.

Reactions

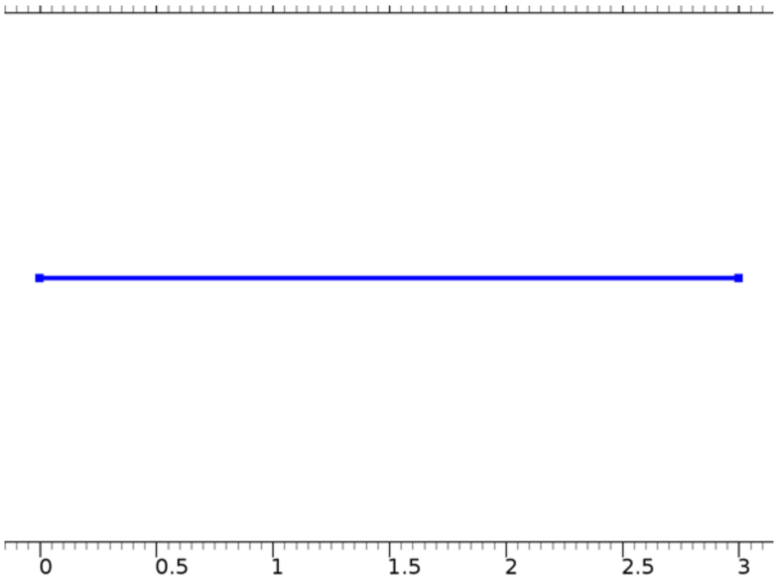

Reactions area

Equatiuons

$$\frac{\partial c_i}{\partial t} + \nabla \cdot (-D_i \nabla c_i) + \mathbf{u} \cdot \nabla c_i = R_i$$

Table S14. Reactions settings

Variable	Reaction R_i
VII	VII_flux
VIIa	VIIa_flux

**Variable**	**Reaction R_i**
V	V_flux + V_e_flux + V_v_flux
Va	Va_flux + Va_e_flux + Va_v_flux
VBe	VBe_flux
VBv	VBv_flux
VaBe	VaBe_flux
VaBv	VaBv_flux
VIII	VIII_flux + VIII_e_flux + VIII_v_flux
VIIIa	VIIIa_flux + VIIIa_e_flux + VIIIa_v_flux
VIIIBe	VIIIBe_flux
VIIIBv	VIIIBv_flux
VIIIaBe	VIIIaBe_flux
VIIIaBv	VIIIaBv_flux
X	X_flux + X_e_flux + X_v_flux
Xa	Xa_flux + Xa_e_flux + Xa_v_flux
IX	IX_flux
IXa	IXa_flux + IXa_e_flux + IXa_v_flux
IXaVIIIae	IXaVIIIae_flux
IXaVIIIav	IXaVIIIav_flux
XIXaVIIIae	XIXaVIIIae_flux
XIXaVIIIav	XIXaVIIIav_flux
PLe	PLe_flux
PLv	PLv_flux
TFPI	TFPI_flux
XaTFPI	XaTFPI_flux
II	II_flux + II_e_flux + II_v_flux
IIa	IIa_flux + IIa_e_flux + IIa_v_flux
IIBe	IIBe_flux
IIBv	IIBv_flux
XaVae	XaVae_flux
XaVav	XaVav_flux
PSVae	PSVae_flux
PSVav	PSVav_flux
IIXaVae	IIXaVae_flux
IIXaVav	IIXaVav_flux
Fg	Fg_flux
Fn	Fn_flux
IIaFg	IIaFg_flux
IIaFn	IIaFn_flux
AT	AT_flux
HC	HC_flux
ATHC	ATHC_flux
ATHCweak	ATHCweak_flux
XI	XI_flux + XI_e_flux + XI_v_flux
XIa	XIa_flux + XIa_e_flux + XIa_v_flux
PS	PS_flux + PS_e_flux + PS_v_flux
a2M	a2M_flux

Variable	Reaction R_i
S	S_flux
IIaS	IIaS_flux
AMC	AMC_flux
IIaa2M	IIaa2M_flux
IIaa2MS	IIaa2MS_flux
XaPS	XaPS_flux
XPS	XPS_flux
XIVBe	XIVBe_flux
XIVBv	XIVBv_flux
XIVaBe	XIVaBe_flux
XIVaBv	XIVaBv_flux

Diffusional transport

Equations

$$\frac{\partial c_i}{\partial t} + \nabla \cdot (-D_i \nabla c_i) + \mathbf{u} \cdot \nabla c_i = R_i$$

$$\mathbf{N}_i = -D_i \nabla c_i + \mathbf{u} c_i$$

Table S15. Diffusion settings

Variable	Diffusion coefficient D_i
VII	D_VII
VIIa	D_VIIa
V	D_V
Va	D_Va
VBe	0
VBv	0
VaBe	0
VaBv	0
VIII	D_VIII
VIIIa	D_VIIIa
VIIIBe	0
VIIIBv	0
VIIIaBe	0
VIIIaBv	0
X	D_X
Xa	D_Xa
IX	D_IX
IXa	D_IXa
IXaVIIIae	0
IXaVIIIav	0
XIXaVIIIae	0
XIXaVIIIav	0
PLe	0
PLv	0
TFPI	D_TFPI
XaTFPI	D_XaTFPI

Variable	Diffusion coefficient D_i
II	D_II
IIa	D_IIa
IIBe	0
IIBv	0
XaVae	0
XaVav	0
PSVae	0
PSVav	0
IIXaVae	0
IIXaVav	0
Fg	D_Fg
Fn	0
IIaFg	D_Fg
IIaFn	0
AT	D_AT
HC	D_HC
ATHC	D_ATHC
ATHCweak	D_ATHC
XI	D_XI
XIa	D_XIa
PS	D_Xa
a2M	D_a2M
S	1e-9[m^2/s]
IIaS	D_IIa
AMC	1e-9[m^2/s]
IIaa2M	D_a2M
IIaa2MS	D_a2M
XaPS	D_XaPS
XPS	D_XaPS
XIVBe	0
XIVBv	0
XIVaBe	0
XIVaBv	0

Influx from the boundary (from TF surface)

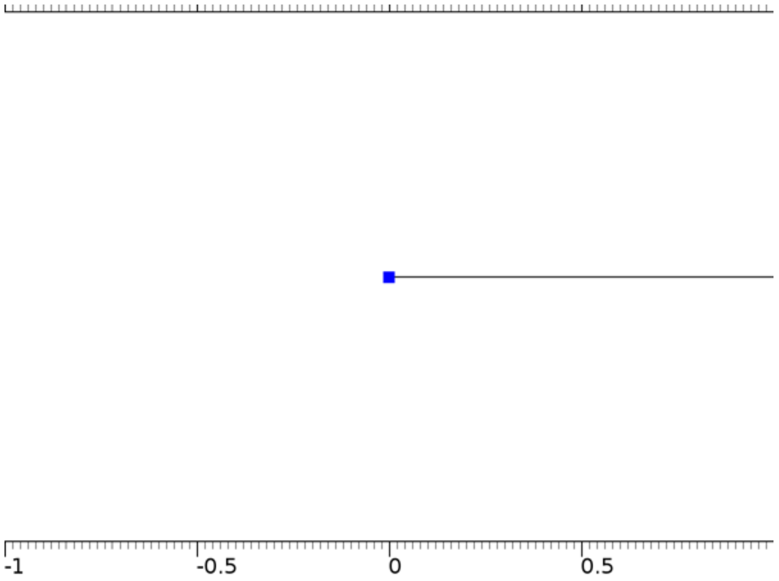

Equations

$$-\mathbf{n} \cdot \mathbf{N}_i = N_{0,i}$$

Table S16. Boundary settings

Variable	Value N_{0i}
VII	sVII_flux
VIIa	sVIIa_flux
V	V_i_flux
Va	Va_i_flux
VBe	0
VBv	0
VaBe	0
VaBv	0
VIII	VIII_i_flux
VIIIa	VIIIa_i_flux
VIIIBe	0
VIIIBv	0
VIIIaBe	0
VIIIaBv	0
X	sX_flux + X_i_flux
Xa	sXa_flux + Xa_i_flux
IX	sIX_flux
IXa	sIXa_flux
IXaVIIIae	0
IXaVIIIav	0
XIXaVIIIae	0
XIXaVIIIav	0
PLe	0
PLv	0
TFPI	0

**Variable**	**Value N_{0i}**
XaTFPI	sXaTFPI_flux
II	II_i_flux
IIa	IIa_i_flux
IIBe	0
IIBv	0
XaVae	0
XaVav	0
PSVae	0
PSVav	0
IIXaVae	0
IIXaVav	0
Fg	0
Fn	0
IIaFg	0
IIaFn	0
AT	0
HC	0
ATHC	0
ATHCweak	0
XI	XI_i_flux
XIa	XIa_i_flux
PS	PS_i_flux
a2M	0
S	0
IIaS	0
AMC	0
IIaa2M	0
IIaa2MS	0
XaPS	0
XPS	0
XIVBe	0
XIVBv	0
XIVaBe	0
XIVaBv	0

Surface reactions

Reaction area

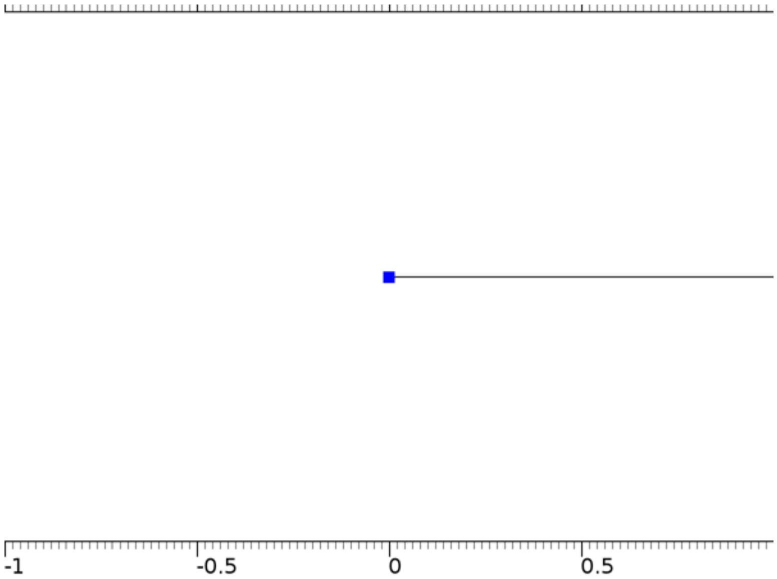

Equations

$$\frac{\partial c_{s,i}}{\partial t} = R_{s,i}$$

Table S17. Boundary reactions settings

**Variable**	**Value R_{si}**
TF	sTF_flux
VIIaTF	sVIIaTF_flux
VIITF	sVIITF_flux
PLi	sPL_flux
XVIIaTF	sXVIIaTF_flux
IXVIIaTF	sIXVIIaTF_flux
XaTFPIVIIaTF	sXaTFPIVIIaTF_flux
VBi	VBi_flux
VaBi	VaBi_flux
VIIIBi	VIIIBi_flux
VIIIaBi	VIIIaBi_flux
IXaVIIIai	IXaVIIIai_flux
XIXaVIIIai	XIXaVIIIai_flux
IIBi	IIBi_flux
XaVai	XaVai_flux
IIXaVai	IIXaVai_flux
XIVBi	XIVBi_flux
XIVaBi	XIVaBi_flux
PSVai	PSVai_flux

Table S18. APTT, PT, anti-Xa activity, antithrombin level, parameters of thrombodynamics-4d 3 hours after LMWH administration (point 1).

Patient	APTT (s)	PT (s)	anti-Xa IU/ml	AT (%)	Tlag (min)	Vi (µm/min)	Vst (µm/min)	A (nM)
1	37.4	12.9	0.375	56	0.9	71.2	30.7	48.3
2	38.2	14.1	0.34694	49	1.2	64.9	27.6	52.2
3	35.1	13.9	0.50909	55	0.9	64.9	27.3	33.7
4	30.6	12.1	0.34677	124	1.3	64.3	28.4	23.7
5	38.7	11.9	0.33019	106	1.1	65.4	24.9	19.6
6	44.7	24.2	1.5102	49	1	48.1	5.5	9.4
7	31.4	15.4	0.18182	66	1.1	73.7	34.5	43.4
8	27.8	12.6	0.23077	52	0.9	83.8	45.6	190.7
9	30.1	18.1	0.27273	44	1.2	67.9	29.1	30.7
10	30.6	14.4	0.5641	78	1.2	60.9	23.3	17.9
11	53.9	13.3	1.28205	78	1	37	5.6	8.8
12	34.1	12.8	0.42697	89	0.9	66.5	26.2	14.3

Table S19. APTT, PT, anti-Xa activity, antithrombin level, parameters of thrombodynamics-4d 6 hours after LMWH administration (point 2)

Patient	APTT (s)	PT (s)	anti-Xa IU/ml	AT (%)	Tlag (min)	Vi (µm/min)	Vst (µm/min)	A (nM)
1	35.9	13.1	0.23636	55	0.9	81.6	42.6	149.7
2	38	14	0.25	52	1.1	64.4	28.7	50.4
3	22.6	11.4	0.29213	89	1.1	66.4	29.9	25.1
4	30.8	12.9	0.2844	109	1.1	67.6	27.7	20.1
5	35.6	11.8	0.19469	113	1.3	66.8	29.7	24.7
6	45.1	25.7	1.44	50	1.4	47.5	5.2	11.9
7	28.7	14.8	0.07042	71	0.8	81.9	78.5	280.5
8	26.4	12.4	0.09434	53	0.9	92.7	84.7	360
9	29.6	16.5	0.15254	59	1.1	69.9	31.3	47.8
10	28.8	13.3	0.41975	81	1.1	68	28.3	39.5
11	53.8	13.9	1.18293	82	1.1	36.7	7.5	8.2

12	35.6	12.9	0.43023	86	0.9	62.8	19.4	52.5

Table S20. APTT, PT, anti-Xa activity, antithrombin level, parameters of thrombodynamics-4d 12 hours after LMWH administration (point 3)

Patient	APTT (s)	PT (s)	anti-Xa IU/ml	AT (%)	Tlag (min)	Vi (μm/min)	Vst (μm/min)	A (nM)
1	29.9	12.3	0.08621	58	0.9	81.2	49.4	254.2
2	34.8	13.9	0.07273	55	1.5	67.2	35.6	134
3	36.6	13	0.22105	95	1	68.8	32.3	23.9
4	34.9	13.7	0.2521	119	1.1	70.1	28.6	13
5	30.6	12.3	0.08824	102	1.1	73.7	42.1	122.3
6	--	--	--	--	--	--	--	--
7	22.6	14.6	0.01493	67	1	81.1	79	263.6
8	23.1	11.9	0.03448	58	0.7	90.3	87.4	473.3
9	34	16.4	0	63	1.3	70.6	39.1	134.5
10	27.7	12.9	0.15068	73	1.1	77.7	40	79.5
11	36.2	13.1	0.55696	79	1.3	57.2	15.5	31.2
12	--	--	--	--	--	--	--	--

Equations of the PK model of LMWH

$$\frac{dC_i}{dt} = -K_{in} \cdot C_i + \frac{V_b}{V_{\text{int}}} \cdot K_{out} \cdot C_{b1} \quad \text{(eq1)}$$

$$\frac{dC_{b1}}{dt} = K_{in} \cdot \frac{V_{\text{int}}}{V_b} \cdot C_i - K_{out} \cdot C_{b1} - Ke\lim \cdot C_{b1} - Ka \cdot C_{b1} + Kd \cdot C_{b2} \quad \text{(eq2)}$$

$$\frac{dC_{b2}}{dt} = Ka \cdot C_{b1} - Kd \cdot C_{b2} \quad \text{(eq3)}$$

C_i is the concentration of LMWH in the interstitial space

C_{b1} is the concentration of the free LMWH in the blood

C_{b2} is the concentration of the bound LMWH in the blood

Table S21. Constant values

Kin	0.0002[1/s]
Kout	0.0001[1/s]

Vb	$1.4\cdot W\cdot 0.075\cdot(1-HC)$ [l]*
Vint	$\frac{W}{80}\cdot 6$ [l]
Kelim	$\left(1+\frac{33}{Creat}\right)\cdot 3.85\cdot 10^{-5}$ [1/s]¶
Ka	$Chol\cdot 0.15\cdot 10^{-5}$ [1/s]Δ
Kd	1e-6 [1/s]

* for male; for female Vb=$1.4\cdot W\cdot 0.065\cdot(1-HC)$; here W is the body weight (kg), HC is the hematocrit (%).

¶ Creat is the creatinine level (μmol/l)

Δ Chol is the total cholesterol level (mmol/l)

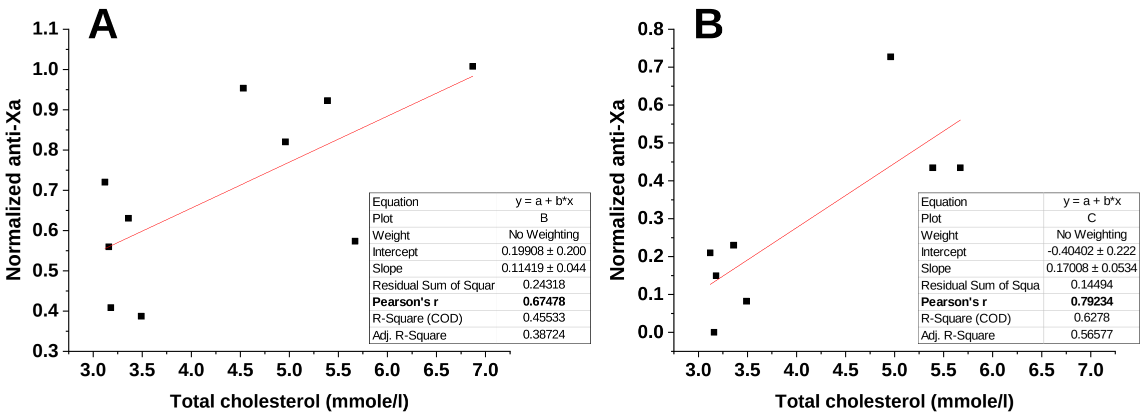

Fig.S1. Correlation of normalized anti-Xa activity and total cholesterol level in the group of patients. A – anti-Xa activity was measured at time point 2 (6 hours after LMWH administration). B - anti-Xa activity was measured at time point 3 (12 hours after LMWH administration).
